# Supplementary material for: Unusual features and localization of the membrane kinome of Trypanosoma brucei
Source: PLoS One. 2021 Oct 15;16(10):e0258814. doi: 10.1371/journal.pone.0258814 (PMC8519429; doi:10.1371/journal.pone.0258814)
Supplement: S1 Fig — The C-terminal PK domain is not shown. Red ovals–transmembrane domains predicted by TMHMM and CCTOP. Each bar below the gene model represents the top hits by HHpred. The color of the arrows mark descriptions matching the hits. The description includes the PDB number and chain designation (Hit), a description of the PDB entry including related PDB entries (Name), the probability of the hit based on the Hidden Markov Model (Probability), the probability of the match in an unrelated database (E-value), score for the secondary structure prediction (SS), number of amino acids aligned (Col), and the total length of the target in PDB (Target Length). The documentation for HHpred considers Probability the most important criterion with positive hits meeting at least one of these criteria: having a score >95% or having a score >50% and making reasonable biological sense [33]. T. cruzi and L. major orthologues have the same predicted folds. (PDF) [file pone.0258814.s001.pdf]

Tb927.11.14070 RDK1 aa 1-912

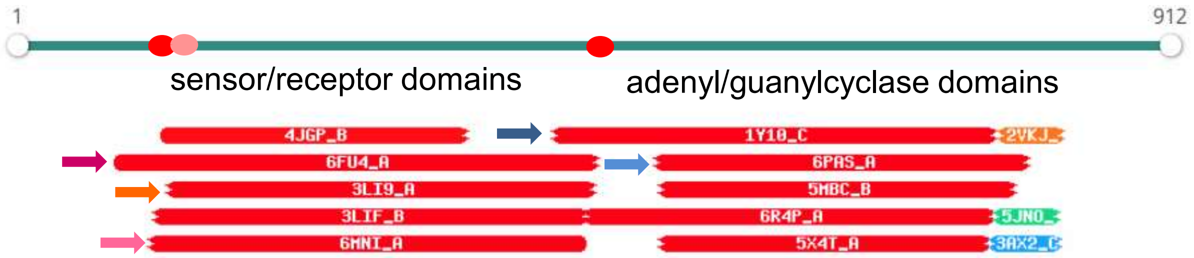

Nr	Hit	Name	Probability	E-value	SS	Cols	Target Length
1	1Y10_C	Hypothetical protein Rv1264/MT1302 (E.C.4.6.1.1); adenylyl cyclase fold, LYASE; HET: 1PE; 2.3A {Mycobacterium tuberculosis}; Related PDB entries: 1Y10_B 1Y10_D 1Y10_A 1Y11_A	99.38	5e-11	23.9	277	407
2	6FU4_A	Chemotaxis protein; chemotaxis, Pseudomonas aeruginosa, signaling protein; HET: GOL, HSM, PO4, MSE; 2.45A {Pseudomonas aeruginosa PAO1}; Related PDB entries: 6FU4_B 6FU4_D 6FU4_C	99.28	1.8e-9	27.6	319	345
3	6PAS_A	Soluble guanylyl cyclase alpha-1 subunit; Nitric oxide, cyclase, H-NOX, SIGNALING; HET: HEM; 5.1A {Manduca sexta}; Related PDB entries: 6PAT_A	99.27	2.6e-10	22.9	243	699
4	3LI9_A	Hypothetical sensory transduction histidine kinase; PDC fold, SIGNALING PROTEIN; HET: MSE, BTB; 1.7A {Methanosarcina mazei} SCOP: d.110.6.4; Related PDB entries: 3LIB_B 3LIB_E 3LIB_J 3LIB_H 3LIB_F 3LIB_D 3LIB_G 3LIB_C 3LIB_I 3LIB_A 3LI8_A 3LIA_B 3LIA_A	99.23	5.5e-9	27.1	276	291
5	3LIF_B	Putative diguanylate cyclase (GGDEF) with; PDC fold, SIGNALING PROTEIN; HET: MSE, MPD, CIT; 2.7A {Rhodopseudomonas palustris}; Related PDB entries: 3LIF_A	99.2	1.8e-8	29.2	241	254
