# Supplementary material for: Unusual features and localization of the membrane kinome of Trypanosoma brucei
Source: PLoS One. 2021 Oct 15;16(10):e0258814. doi: 10.1371/journal.pone.0258814 (PMC8519429; doi:10.1371/journal.pone.0258814)
Supplement: S2 Fig — Parasites were grown in the absence of Tet or in the presence of Tet to induce the V-5 tagged PK. Two independent clonal isolates are shown for FHK. Clone 2 was used for the localization data shown in Fig 8. The migration of each tagged protein is marked. The predicted molecular weights for each protein are provided in S1 Table. (PDF) [file pone.0258814.s002.pdf]

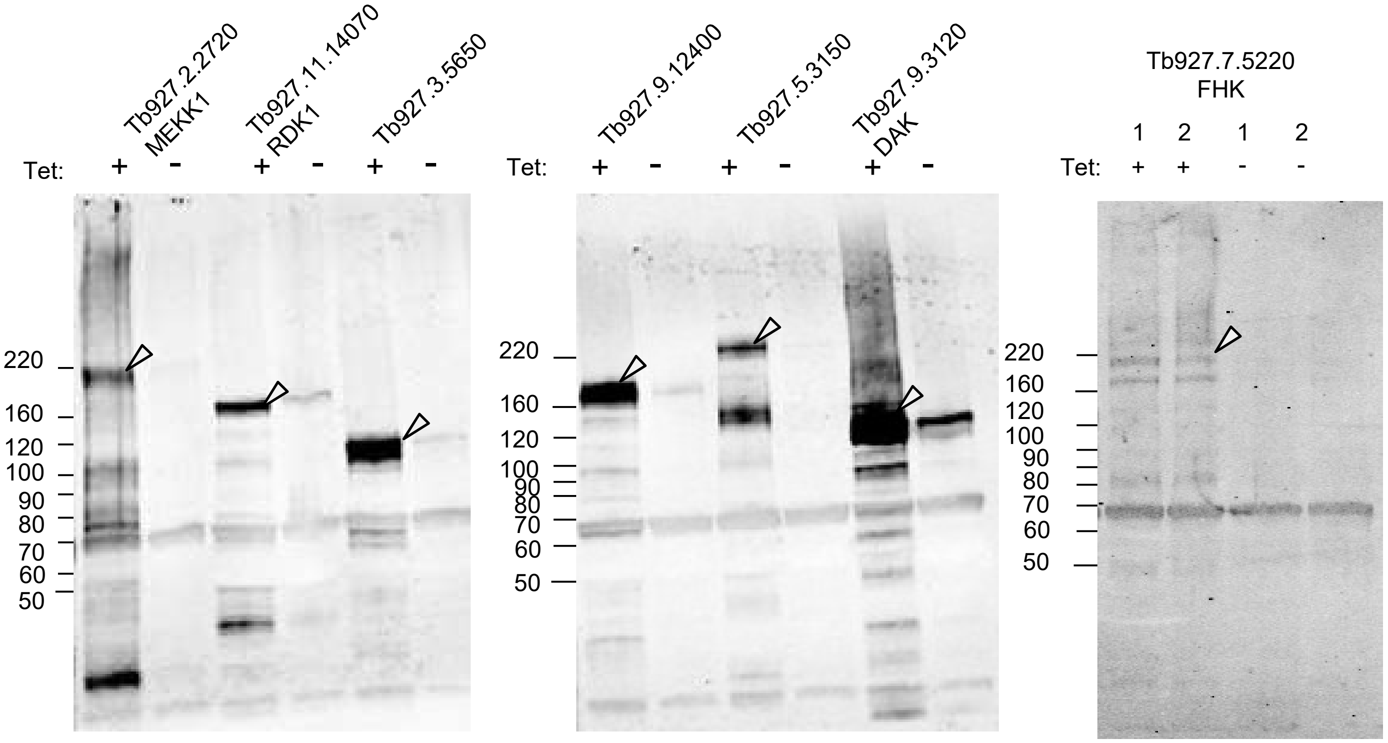

Figure S1. Western blot showing expression of epitope-tagged PKs in *T. brucei* bloodstream forms. Parasites were grown in the absence of Tet or in the presence of Tet to induce the V-5 tagged PK. Two independent clonal isolates are shown for FHK. Clone 2 was used for the localization data shown in Fig 8. The migration of each tagged protein is marked. The predicted molecular weights for each protein is provided in Table S1. The migration of molecular mass markers was at the left of each blot.
