# Supplementary material for: Unusual features and localization of the membrane kinome of Trypanosoma brucei
Source: PLoS One. 2021 Oct 15;16(10):e0258814. doi: 10.1371/journal.pone.0258814 (PMC8519429; doi:10.1371/journal.pone.0258814)
Supplement: S1 Table — (PDF) [file pone.0258814.s003.pdf]

Table S2- Primers used

**Primer**	**Sequence (5'-3')**
MEKK1-AvrII-ATG	CCGCCTAGGATGCCCTTCGCGGCAAATGTTG
MEKK1-HindII-P4503M	CGGAAGCTTAACAACTCCCTGATCACCAAATG
Tb927.3.5650-AvrII-ATG	CCTAGGATGATAGAATATGCGTGTGGTTG
Tb927.3.5650-BamHI-P2787M	GGATCCCAAATGAAATTCGCTTCTCGAGATATTCCCAC
Tb927.5.3150-AvrII-ATG	CCTAGGATGGTTGTGCGGCCATTGTTCTG
Tb927.5.3150-BamHI-P4962M	GGATCCAAGCTCCTGGACATTACTGAAGAAG
FHK-AvrII-ATG	CGGCCTAGGATGACACAGCCCTCGGCAGATG
FHK-XhoI-P5034M	CGGCTCGAGATTACCTTCCACAGCAGCGTTAC
Tb927.9.12400-AvrII-ATG	CCTAGGATGTGTGCCTTAAGGGATATCGCGTCAAC
Tb927.9.12400-BglII-P4035M	AGATCTGTTTACCATAAAGTCATGGAACAAAAGCTCAG
DAK-AvrII-ATG	CCGCCTAGGATGGGACCAACATGTGATCGAATAG
DAK-BamHI-P3039M	CGCGGATCCCGAATGGGGTTGTGTGGTGATGGTAAG
RDK1-AvrII-ATG	CCGCCTAGGATGACGAAAGAGGATCACGGTG
RDK1-BamHI-P3585M	CGCGGATCCAACAAAAATGCATATGAAAGTAAACTGGATG

Underlined bases are those used for inserting the gene into pT7-3V5-Hyg. Primer names include the restriction sites used for cloning. Bases preceding the restriction site were added to facilitate restriction digest of the PCR fragments.
