# Supplementary material for: Unusual features and localization of the membrane kinome of Trypanosoma brucei
Source: PLoS One. 2021 Oct 15;16(10):e0258814. doi: 10.1371/journal.pone.0258814 (PMC8519429; doi:10.1371/journal.pone.0258814)
Supplement: S1 Raw images — (PDF) [file pone.0258814.s005.pdf]

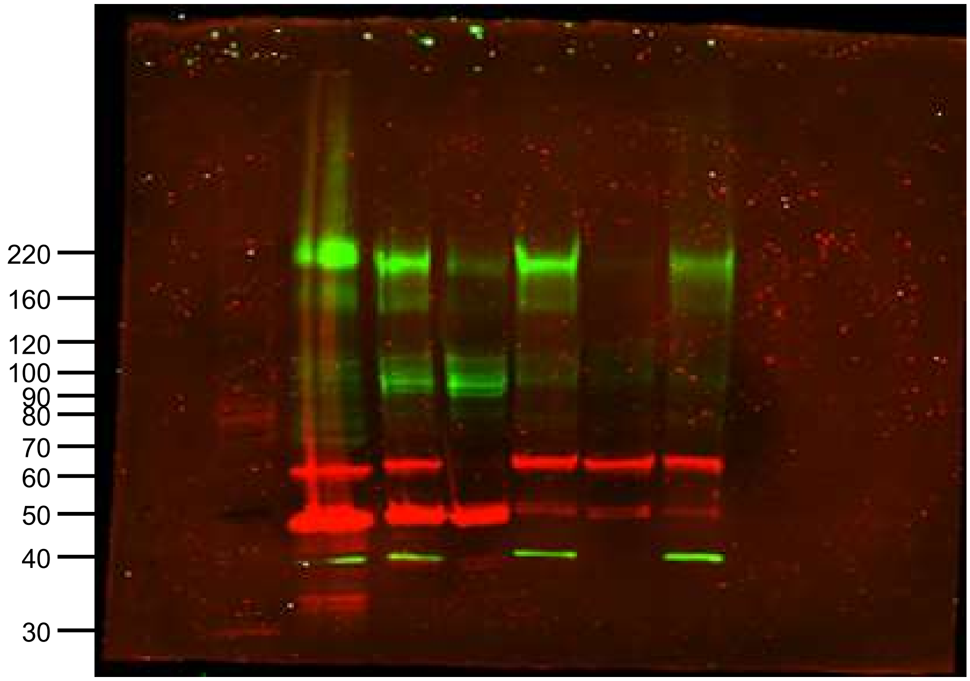

Fig 7A upper panel-western blot of cell fractionation in PF cells expressing MEKK1-HA. Lanes L) Ladder, SDS) SDS lysate; T) total after incubation with digitonin, S1) digitonin supernatant, P1) digitonin pellet, S2) carbonate supernatant, P2) carbonate pellet. Membrane was simultaneously probed with mouse α-HA (green bands) and rabbit α-PGK (red bands). Primary antibodies were detected with either IR680 dye conjugated goat α-rabbit or IR800 dye conjugated goat α-mouse. The membrane was imaged using the Licor Odyssey.

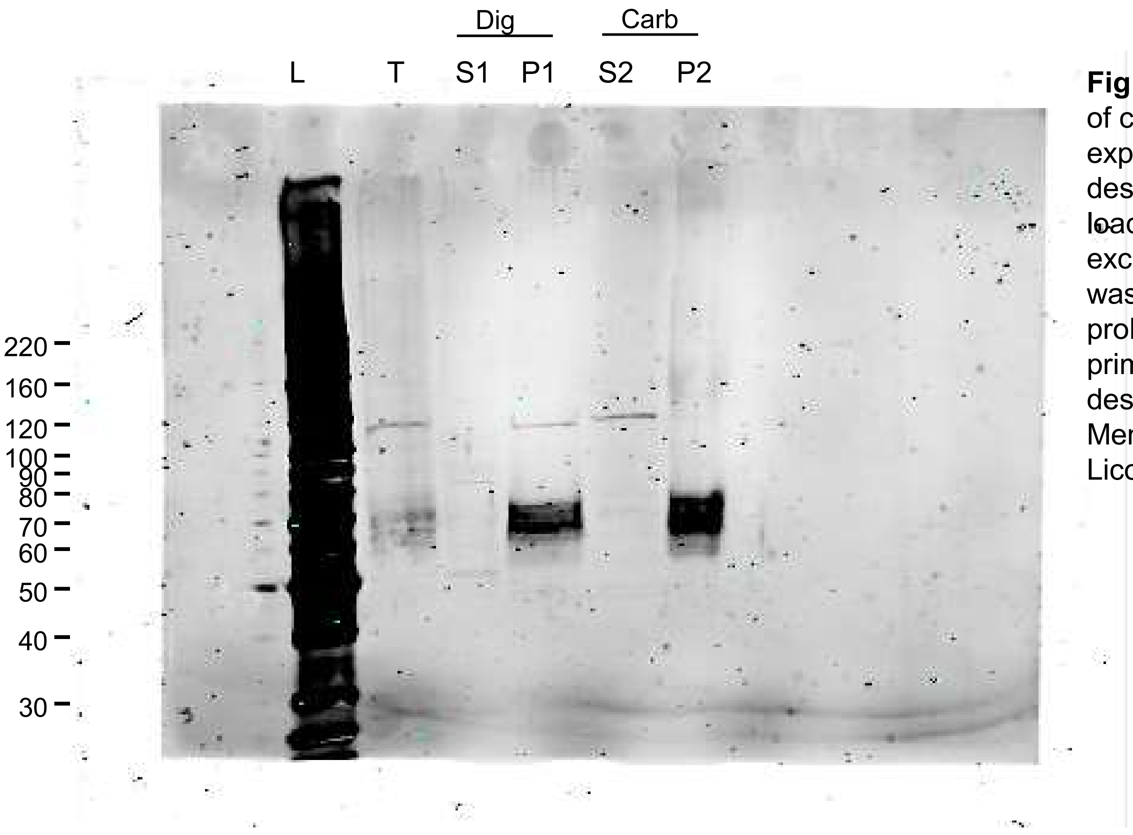

Fig 7A lower panel-western blot of cell fractionation in PF cells expressing MEKK1-HA. Samples described for upper panel were loaded in the same order with the exception that the SDS sample was omitted. The membrane was probed with rabbit α-VTC with the primary antibody detected as described for upper panel. Membrane was imaged using the Licor Odyssey.

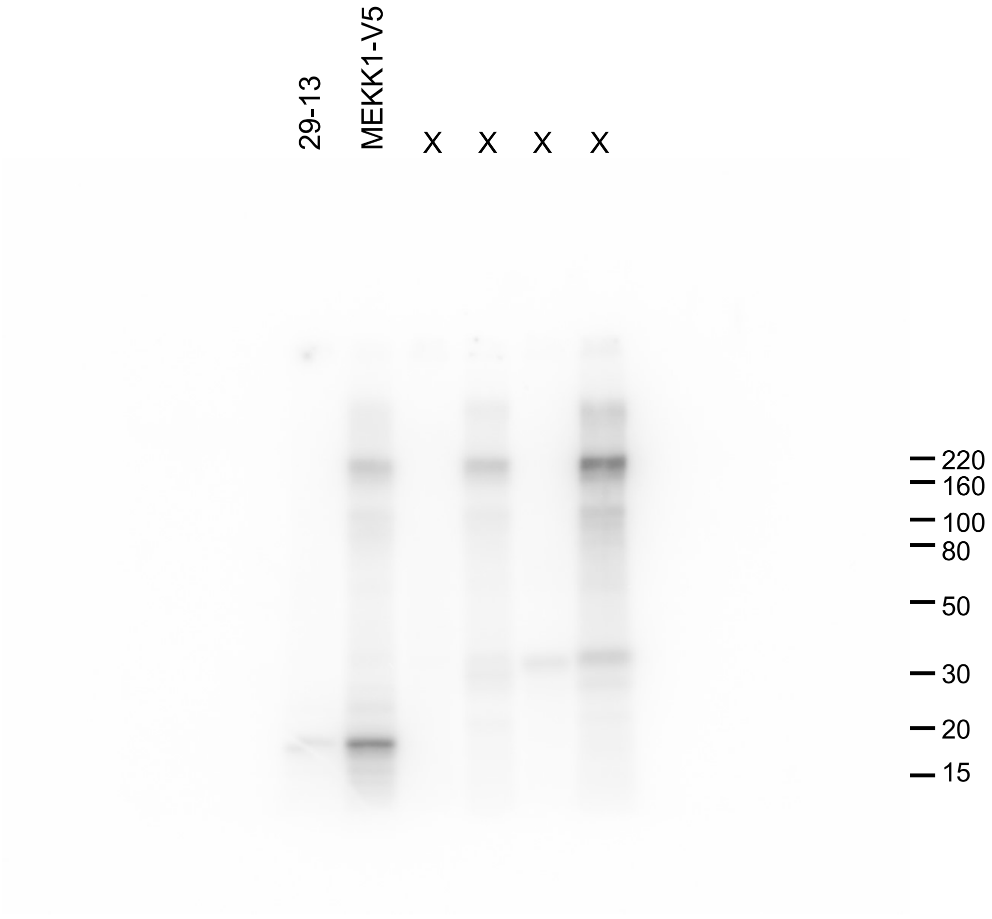

Fig 7B Top panel-Phosphorimage of IP-kinase assay from either untransfected PF cells (29-13) or cells expressing V5 tagged MEKK1 (MEKK1-V5) with MBP as substrate. Image was collected on Storm Imager (GE) Note: image in Fig 5 is flipped right to left.

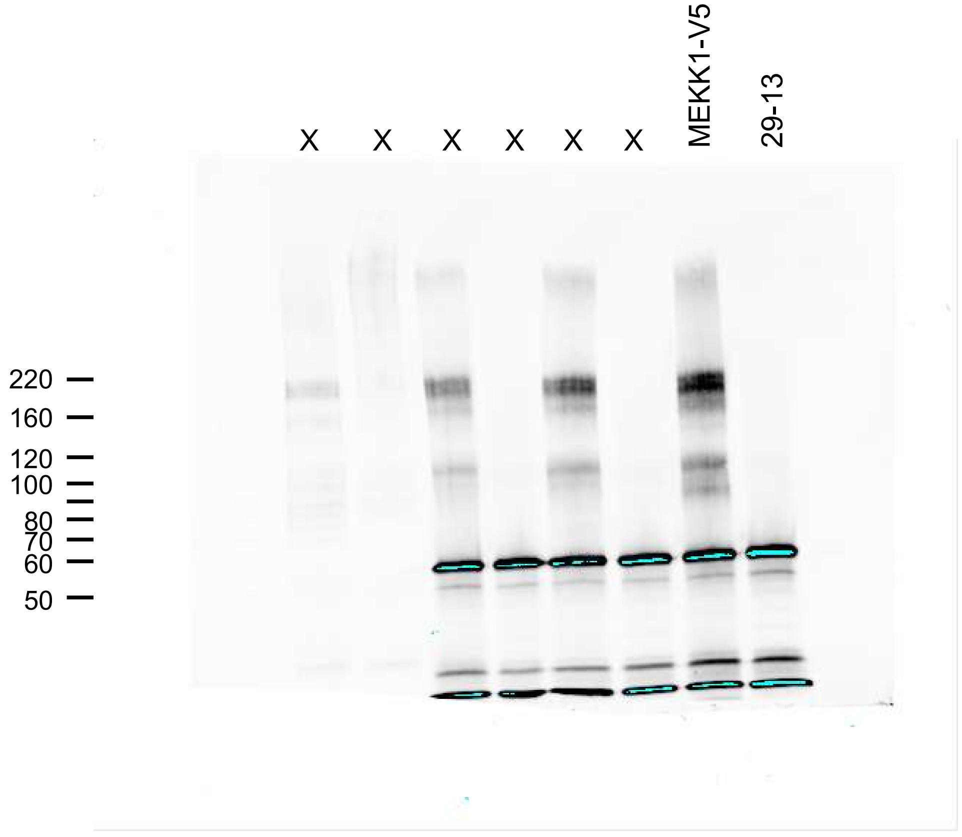

Fig 7B bottom panel-western blot of IP-kiinase assay from either untransfected PF cells (29-13) or cells expressing V5 tagged MEKK1 (MEKK1-V5).Membrane was probed with mouse α-V5 with primary antibody detected with IR800 dye conjugated goat α-mouse. Membrane was imaged using the Licor Odyssey. Note: image in Fig 5 is flipped right to left.

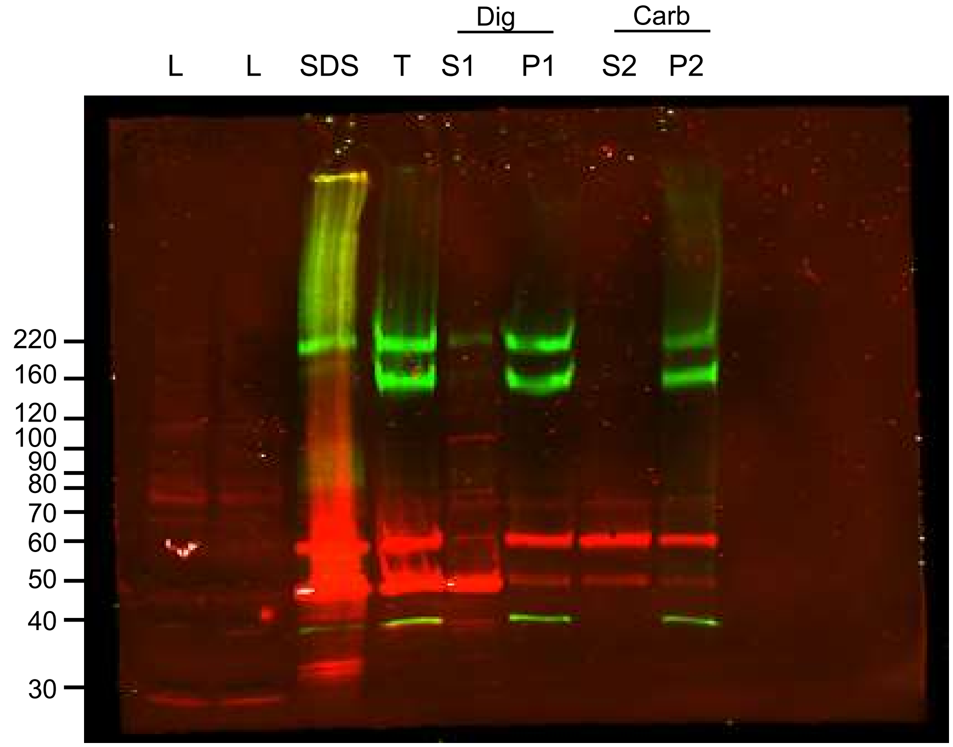

Fig 10A upper panel-western blot of cell fractionation in PF cells expressing FHK-V5. Lanes L) Ladder, SDS) SDS lysate; T) total after incubation with digitonin, S1) digitonin supernatant, P1) digitonin pellet, S2) carbonate supernatant, P2) carbonate pellet. Membrane was simultaneously probed with mouse α-V5 (green bands) and rabbit α-PGK (red bands). Primary antibodies were detected with either IR680 dye conjugated goat α-rabbit or IR800 dye conjugated goat α-mouse. The membrane was imaged using the Licor Odyssey.

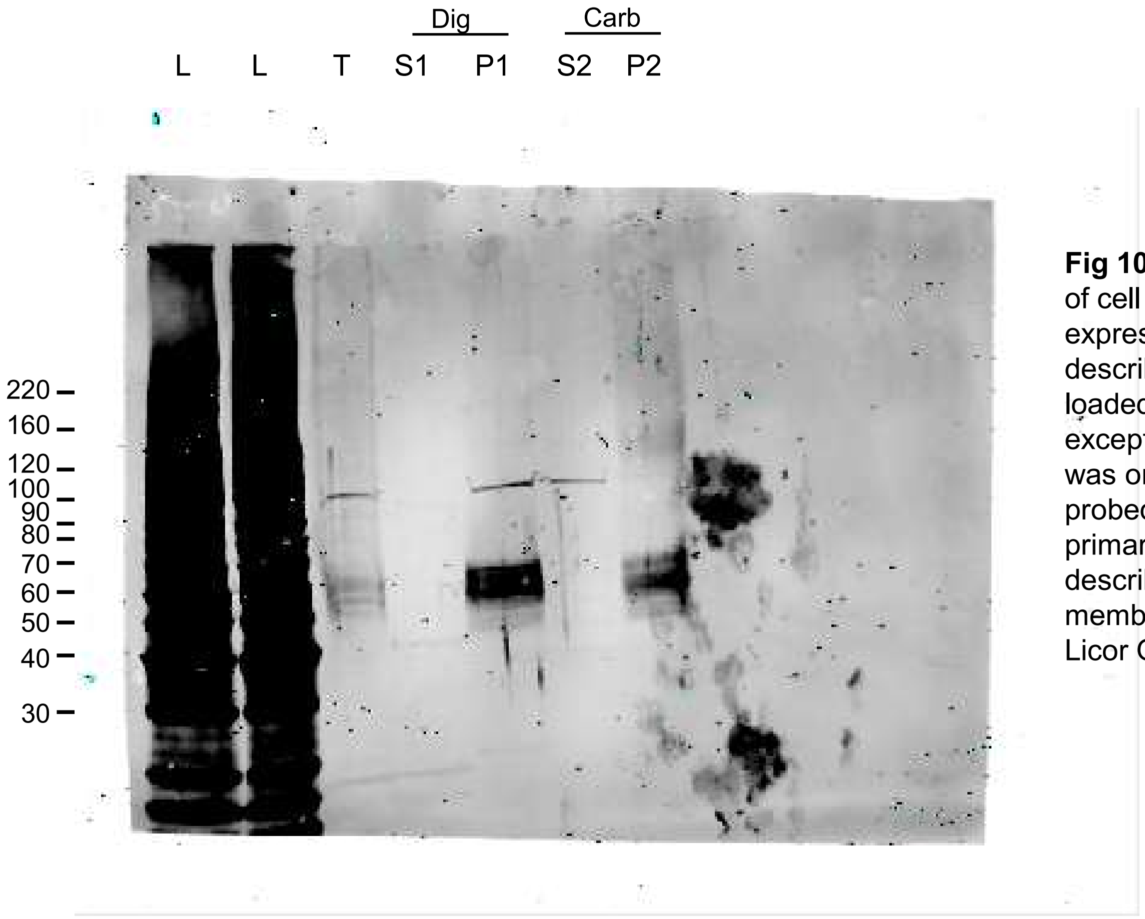

Fig 10A lower panel-western blot of cell fractionation in PF cells expressing FHK-V5. Samples described for upper panel were loaded in the same order with the exception that the SDS sample was omitted. Membrane was probed with rabbit α-VTC with the primary antibody detected as described for upper panel. . The membrane was imaged using the Licor Odyssey.

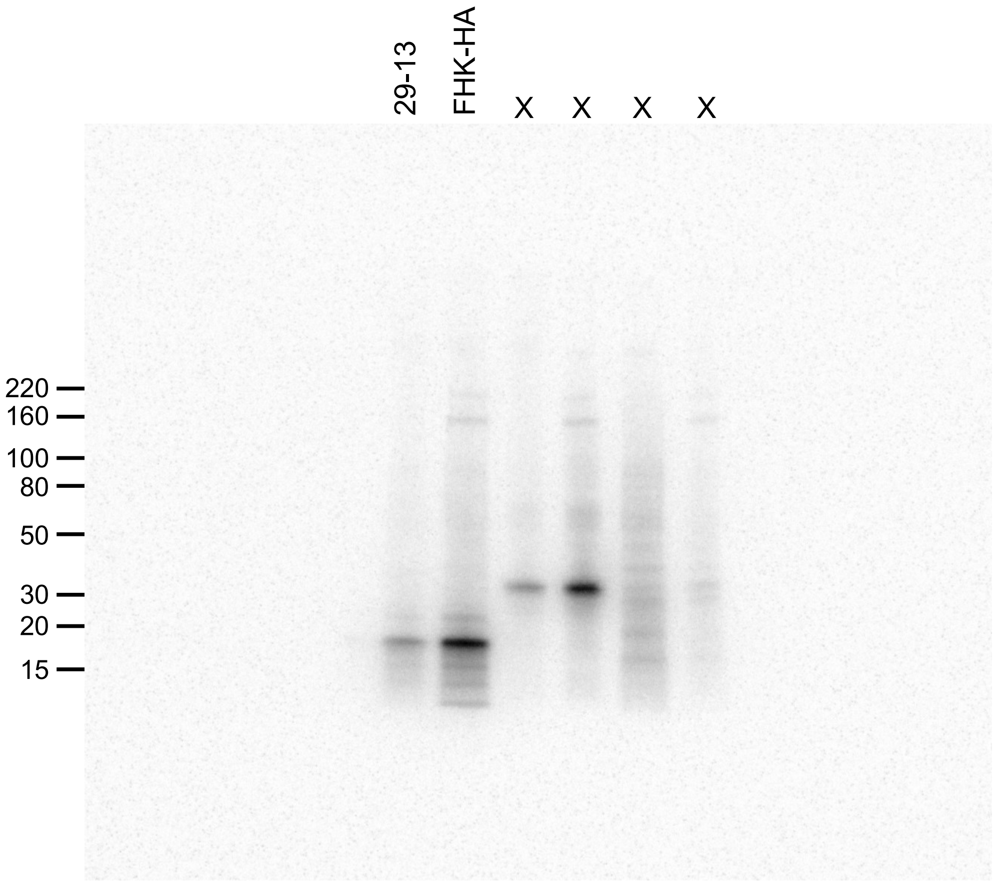

Fig 10B Top panel-phosphorimage of IP-kinase assay from either untransfected PF cells (29-13) or cells expressing HA tagged FHK-HA (FHK-HA) with MBP as substrate. Image was collected on Storm Imager (GE).

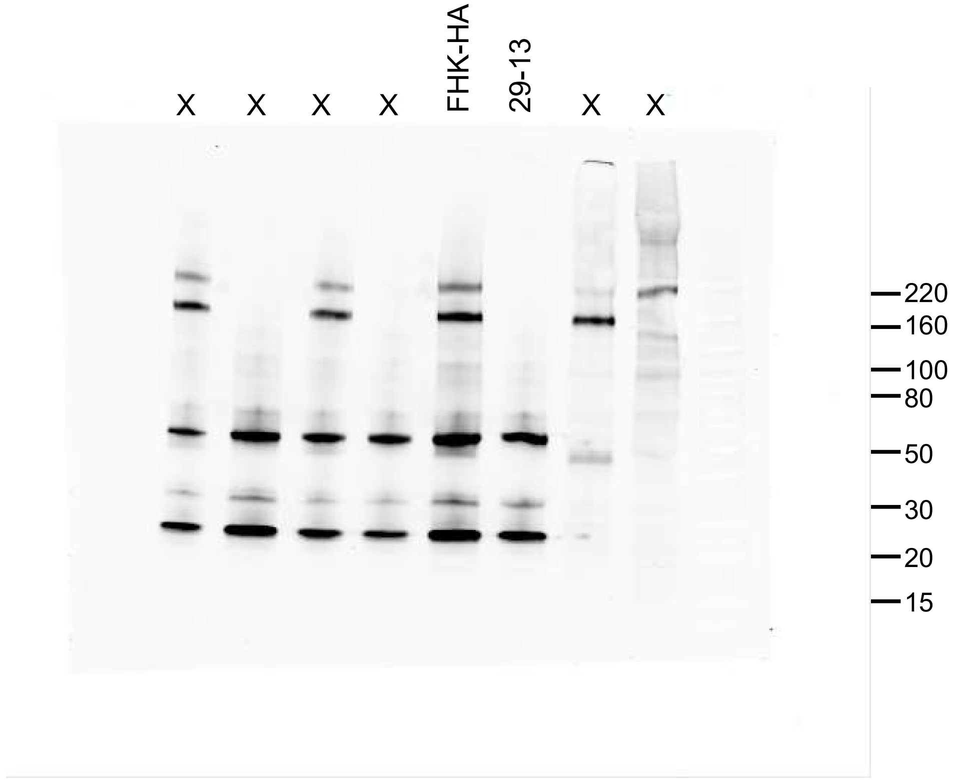

Fig 10B bottom panel-western blot of IP-kiinase assay from either untransfected PF cells (29-13) or cells expressing HA tagged FHK (FHK-HA). Membrane was probed with mouse α-HA with primary antibody detected with IR800 dye conjugated goat α-mouse.. Membrane was imaged using the Licor Odyssey. Note: image in Fig 10 is flipped right to left.

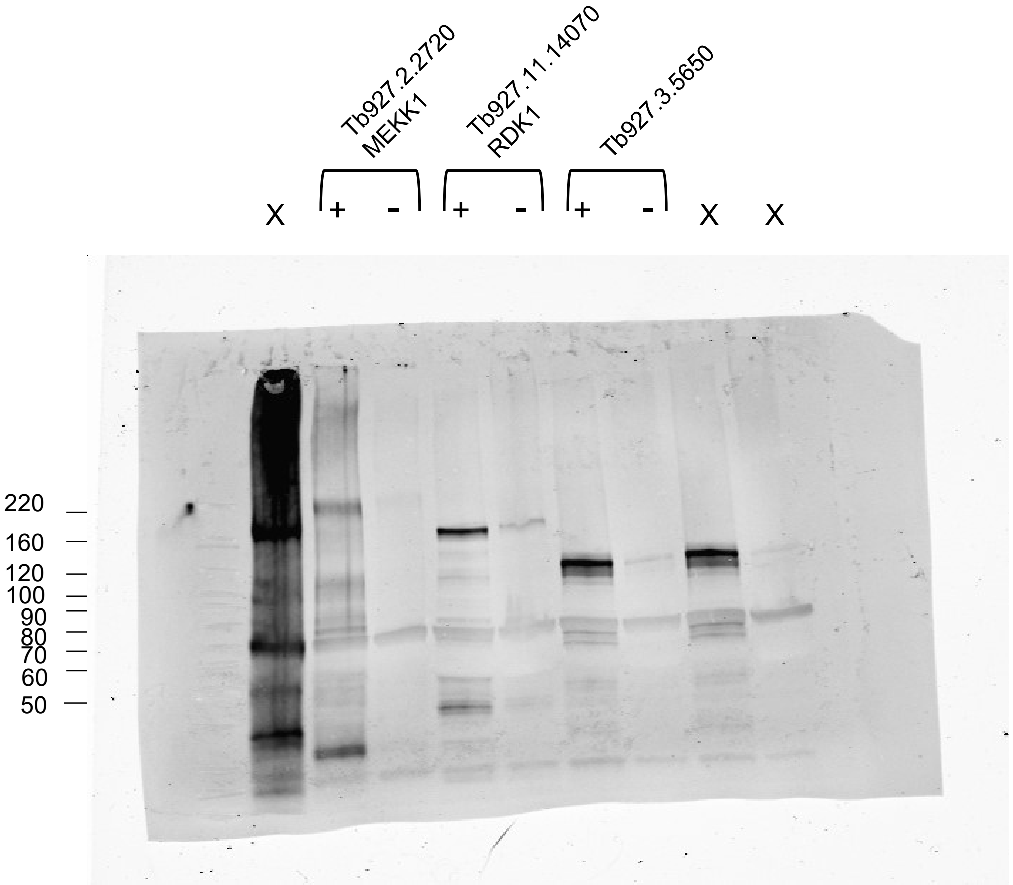

S2 Fig Left panel- Western of V5 tagged proteins used for localization of cell lysates from either induced Tet+ or uninduced Tet- for the indicated gene. The membrane was probed with mouse α-V5 with the α-V5 detected with IR800 dye conjugated goat α-mouse. The membrane was imaged using the Licor Odyssey.

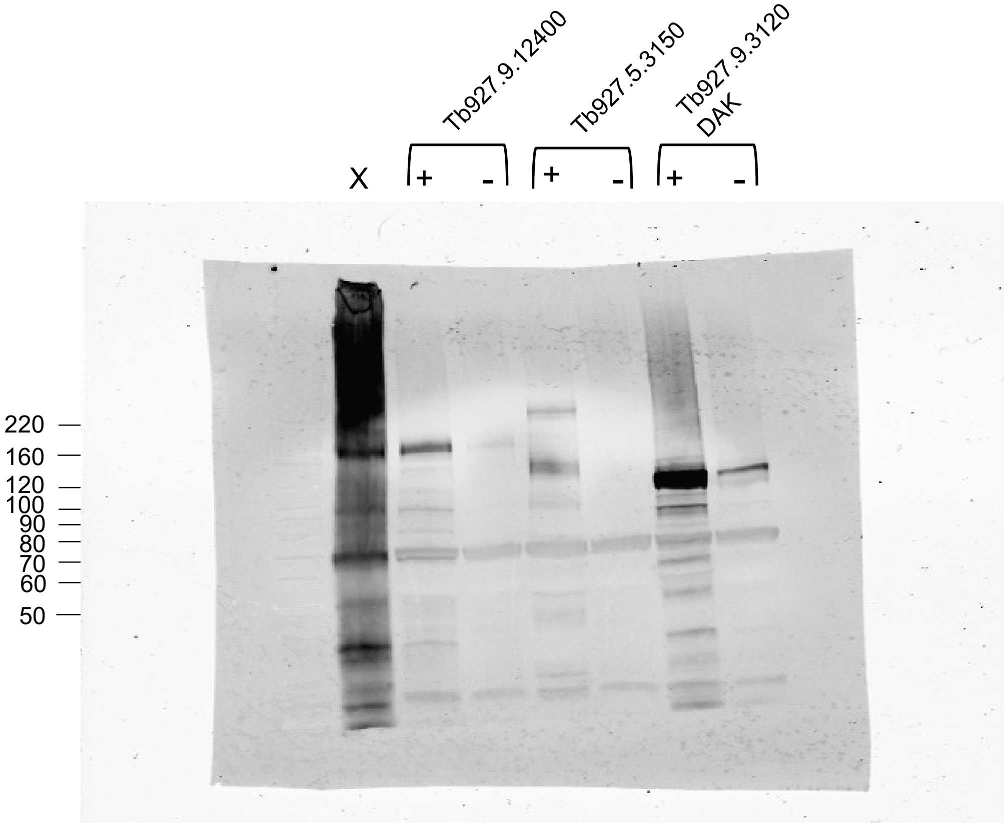

S2 Fig Middle panel- see legend for S2 Left panel above

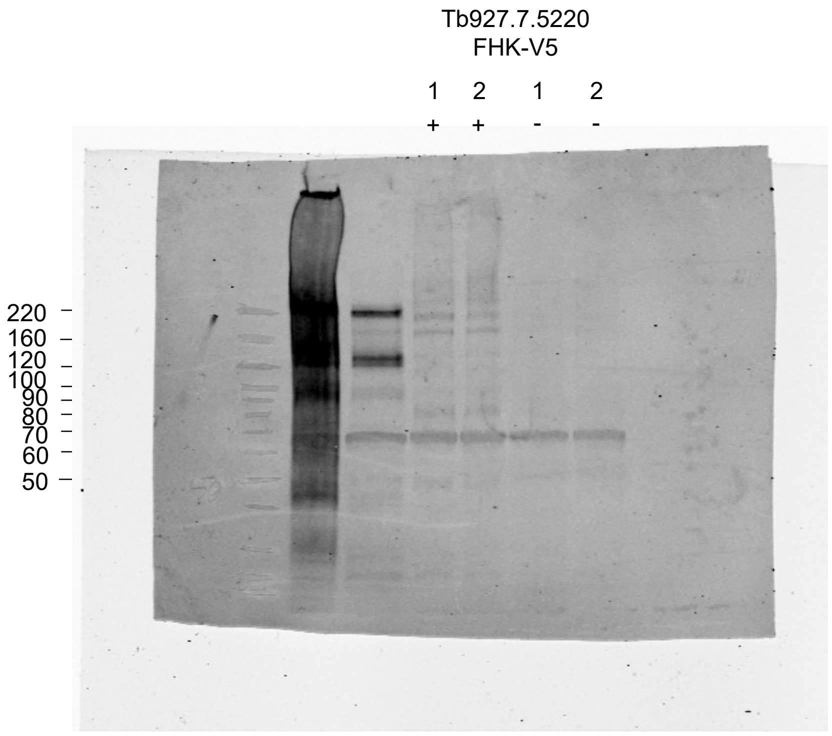

S2 Fig Right panel- SDS lysates from either induced Tet+ (+) or uninduced Tet- (-)from two independent clones expressing V5 epitope tagged FHK. Membrane was probed with mouse α-V5 with IR800 conjugated goat α-mouse as a secondary. Membrane imaged using Licor Odyssey.
