# Supplementary material for: Unusual features and localization of the membrane kinome of Trypanosoma brucei
Source: PLoS One. 2021 Oct 15;16(10):e0258814. doi: 10.1371/journal.pone.0258814 (PMC8519429; doi:10.1371/journal.pone.0258814)
Supplement: Attachment — Submitted filename: Response to reviewers comments.docx [file pone.0258814.s006.docx]

Response to reviewers’ comments on submission to PLoS NTDS

Reviewer #1

The paper provides only a modest increase in knowledge on protein kinases in trypanosomes, as there are no new functional insights into the roles of the kinases in the cell. In particular, the authors identify potential histidine kinase domains, but don’t do any follow up analysis to determine whether they are important to the parasite. We believe that this initial study adds to our knowledge of the small set of *T. brucei* transmembrane kinases. Unfortunately, without considerably more data on the function of these kinases it is difficult to determine the role of specific domains, such as the histidine kinase domains. These PKs, including the one with a histidine kinase domain, appear to be dispensible for parasite growth in vitro and in vivo, and have not been linked to stress resistance or drug resistance. Thus, probing structure/cellular function relationships is not feasible at present. Only one of the PKs we examined has a clearly understood function (MEKK1), which plays a role in differentiation (studied by Matthews group).

1. Colocalization would be strengthened with the quantitative data on localization using Pearson correlation co-efficient. This data was added to Fig 4, with values between 0.56 and 0.76 strongly supporting colocalization.
2. A suitable control experiment would be to run an IFA for a surface protein or other cell membrane protein to show that that location is not disrupted by their fixation conditions This control is included in Fig 5 (the K+ channel. Protein). Additionally, these conditions have been used to study membrane proteins in both bloodstream form and procylic forms (PMIDs 33745971 , 22351763, 15342636).
3. Fixation conditions need to be added to Methods section. They were included in the Methods section. We have added additional details and mention in the legend to Fig 4 that fixation conditions can be found in the Methods.

Reviewer #2

The main issue with the manuscript is that it is the initial characterization of a set of proteins that functions more as a beginning point rather than a completed study. There are no specific data that go towards the function of these proteins, which would be valuable. See response to Reviewer 1 (opening comment).

1. Informatics methods used to predict TMDs should be tested with a set of known proteinsWe used a suite of published software packages that were developed and validated using non-trypanosomal proteins. Relevant references are now included in the manuscript. The main algorithm we used, TMHMM, is also the one that is used by TriTrypDB.
2. Why do the authors use T7 expression system versus endogenous tagging? T7 expression was used to get sufficient expression for localization (noted in the manuscript).
3. It would be important to validate this result biochemically. A protease protection assay of using N- and C-terminally tagged MEKK could be used. This experiment requires that the test protein reside on the plasma membrane (or an intracellular, digitonin-resistant membrane). Of the PKs localized in this manuscript only two were localized with domains at the cell surface with the remaining five localized to interior organelles. Both of the proteins localized to the cell surface and showed instability during cell processing confounding any interpretation, see Figs 7 and Fig 10. ER-localized proteins are unsuitable for these studies since the plasma membrane must be solubilized with digitonin to permit access to intracellular membranes, but the like the plasma membrane, ER is rich in sterols and sensitive to digitonin.
4. Minor issues
   a. Are the same patterns of TMDs predicted in *Leishmania* and *T. cruzi*? Yes, so stated in the text an in Table S1. We have not examined the Bodo CDSs.
   b. Do any of the PKs have an ER retention signal, MDDL for example? MDDL is typically used for retention of soluble proteins rather than membrane proteins and must be localized at the C-terminus to function. The kinases did not bear such sequences. The motifs for retention of membrane proteins are more complex and less well-understood, although some are C-terminal. It is unlikely that a C-terminal sequence is important for ER retention of these proteins, as it would be masked by the epitope tag.
   c. Figure 4, degree of localization would be helpful. Done, see comment above for reviewer #1.
   d. If the proteins do not remain in the ER where do they go? The authors could non-permeabilizing or minimally permeabilizing fixations to see if they can see labeling at the cell surface, without allowing access to the ER. Unfortunately, antibodies cannot penetrate the surface VSG coat to access surface proteins without membrane permeabilization, making this experiment problematic.
   e. V5 signal should be green in throughout Fig. 5. We used red for V5 for the top panels, since it has greater contrast against the phase image than green. The panels are clearly labeled so this should not be too confusing.
   f. What structure does the K+ channel antibody label, could it be the FAZ? To our knowledge this antibody had not been used in *T. brucei*. In *T. cruzi* the protein was localized adjacent to the FAZ. This has been added to the text of the manuscript.
   g. The (MEKK1) protein appears to be heavier than expected, correct? The way it’s stated now makes it seem that it should be running above the 220 KD marker. Could phosphorylation of the kinase be causing it to run more slowly than expected? We reworded to more accurately describe the migration of the protein – it does run large but not slower than 220 kDa. Given the size of MEKK1, we would not predict that phosphorylation would have a significant impact on its mobility in SDS-PAGE. As referenced now in the text, the most likely reason is that membrane proteins can run larger than expected.
   h. Fig. 7. Not sure why experiment is included, is activity coming from tagged protein versus an associated protein. Our opinion is that it is better to show activity experimentally than to infer it from sequence. The control shows that kinase activity requires MEKK1 and is not due to a kinase directly adhering to the beads. It is formally possible, albeit unlikely, that the tagged kinase is inactive but interacts with an active kinase responsible for the detected activity.
   i. Fig 9. I don’t see any evidence for vesicular labeling for FHK in 9C or 9D. Vesicles were labeled in 9C (marked by “v”) Since specific vesicles are not distributed evenly throughout the cell, we do not expect to see them in all images. Since the flagellar pocket is not visible in 9D, it is difficult to distinguish labeling of the flagellar pocket from labeling of vesicles. That we see labeling of multiple distinct vesicular like structures, some of these must be vesicle since the cell contains a single flagellar pocket.
   j. Authors should refrain from speculating about the distribution of labeling without quantitation. We have removed this language from the manuscript.
   k. Fig. 10. Same as comment h above.
